# Descripción angiográfica de los aspectos anatómicos y clínicos de la arteria interventricular anterior en un grupo de personas colombianas

**DOI:** 10.7705/biomedica.7080

**Published:** 2023-12-01

**Authors:** Gustavo Pabón, Valentina Patiño, Guillermo Rivera

**Affiliations:** 1 Programa de Medicina, Pontificia Universidad Javeriana, Cali, Colombia Pontificia Universidad Javeriana Programa de Medicina Pontificia Universidad Javeriana Cali Colombia; 2 Departamento de Ciencias Básicas de la Salud, Pontificia Universidad Javeriana, Cali, Colombia Pontificia Universidad Javeriana Departamento de Ciencias Básicas de la Salud Pontificia Universidad Javeriana Cali Colombia

**Keywords:** anomalías de los vasos coronarios, puente miocárdico, enfermedad de la arteria coronaria, angiografía coronaria, dolor en el pecho, coronary vessel anomalies, myocardial bridging, coronary artery disease, coronary angiography, chest pain

## Abstract

**Introducción.:**

La arteria interventricular anterior se origina en la coronaria izquierda, irriga la cara anterior de los ventrículos, el ápex y el tabique interventricular; es la segunda arteria más relevante del corazón.

**Objetivo.:**

Describir las características anatómicas y clínicas de la arteria interventricular anterior mediante angiografía.

**Materiales y métodos.:**

Se realizó un estudio descriptivo con 200 reportes angiográficos de personas colombianas; se valoraron el origen, el trayecto y la permeabilidad de la arteria interventricular anterior, así como la dominancia coronaria. Se incluyeron datos relacionados con dolor precordial, infarto agudo de miocardio, dislipidemia y alteración electrocardiográfica. No fue posible hacer pruebas estadísticas, debido a la escasa prevalencia de variaciones anatómicas de dicha arteria.

**Resultados.:**

Se encontró una arteria interventricular anterior con su origen en el seno aórtico izquierdo, sin puente miocárdico, sin alteración de la permeabilidad y con dominancia izquierda. La frecuencia de los puentes fue del 2 % y la dominancia más frecuente fue la derecha en el 86 %. Se presentaron alteraciones de permeabilidad en el 43 % de los casos, las cuales afectaron principalmente al S13. El 25 % de los pacientes presentó dolor precordial; el 40 %, alteraciones ecocardiográficas; el 5 %, cardiopatía isquémica, y el 59 %, alguna alteración electrocardiográfica.

**Conclusiones.:**

Las variaciones en el origen de la arteria interventricular anterior son poco prevalentes, según reportes de Chile, Colombia y España. Los puentes miocárdicos de esta arteria fueron escasos respecto a otros estudios, lo cual sugiere mejor especificidad de los hallazgos de la angiotomografía o de la disección directa. La permeabilidad coronaria se valora con la escala TIMI (*Thrombolysis in Myocardial Infarction*); puntajes de 0 y 1 indican una lesión oclusiva asociada con cardiopatía isquémica. La dominancia coronaria más frecuente, según diversas técnicas, es la derecha, seguida de la izquierda en hombres y de una circulación balanceada en mujeres.

## Abstract (en)

**Introduction.:**

The anterior interventricular artery originates from the left coronary artery and irrigates the anterior surface of the ventricles, apex, and interventricular septum, making it the second most relevant artery of the heart.

**Objective.:**

To describe the anatomical and clinical aspects of the anterior interventricular artery through angiography.

**Materials and methods.:**

A descriptive study was conducted using 200 angiographic reports of Colombian individuals. The anterior interventricular artery's origin, course, patency, and coronary dominance were evaluated. Data related to chest pain, acute myocardial infarction, dyslipidemia, and electrocardiographic abnormalities were included. Statistical tests could not be performed due to this artery's low prevalence of anatomical variations.

**Results.:**

One anterior interventricular artery was found to have originated from the left coronary sinus without a myocardial bridge, with no alteration in permeability, and with left dominance. The frequency of bridges was 2%, and the most frequent dominance was right in 86; permeability alterations occurred in 43% mainly affecting S13. Twenty-five per cent presented chest pain; 40%, echocardiographic alterations; 5%, ischemic heart disease, and 59%, electrocardiographic alterations.

**Conclusions.:**

Variations of origin of the anterior interventricular artery have a low prevalence according to reports from Chile, Colombia, and Spain. anterior interventricular artery myocardial bridges were scarce compared to other studies, suggesting better specificity of computed tomography angiography or direct dissection for these findings. The assessment of coronary permeability is graded with the thrombolysis in myocardial infarction scale; values 0 and 1 indicate occlusive lesion associated with ischemic heart disease. According to various techniques, the most frequent coronary dominance the right, followed by the left in men and balanced circulation in women.

El conocimiento detallado de los aspectos anatómicos y las consideraciones clínicas de la arteria interventricular anterior es fundamental para el diagnóstico y tratamiento de la enfermedad arterial coronaria que es la tercera causa de morbimortalidad mundial y la primera en Colombia ^1^.

La interventricular anterior es una de las principales arterias que irrigan el corazón; se origina en la arteria coronaria izquierda, discurre por el espacio subepicárdico sobre el surco interventricular anterior, y se divide en los segmentos S12, S13 y S14 ^2^. Emite ramas que irrigan la pared anterior del ventrículo izquierdo, los dos tercios anteriores del tabique interventricular y el ápex cardiaco ^3^.

Las variaciones del origen de la arteria interventricular anterior oscilan entre el 0,6 y el 1,3 %; incluyen su origen directo en el seno aórtico izquierdo o el derecho, en el tronco común con la coronaria derecha y en el tronco pulmonar. Pocas veces son asintomáticas, ya que se relacionan con cardiopatía isquémica e, incluso, muerte súbita ^4^^-^^7^. La permeabilidad de la arteria interventricular anterior se puede valorar mediante una técnica angiográfica. La disminución de la luz arterial se relaciona con dolor precordial; si la obstrucción es igual o superior al 70 %, requiere de intervencionismo, por el riesgo de infarto miocárdico ventricular izquierdo y de bloqueo aurículo-ventricular asociado con lesión del fascículo auriculoventricular, también conocido como haz de His ^8^.

La información disponible en los textos de anatomía descriptiva sobre la arteria interventricular anterior, o arteria descendente anterior izquierda, que se usa como insumo para la formación de estudiantes de pregrado y posgrado en medicina y otras ciencias de la salud, suele limitarse a la descripción del origen anatómico normal y de un solo patrón de ramificación ^9^^,^^10^.

En el presente artículo, se describen aspectos más detallados de variables como el trayecto, la permeabilidad y la relación de esta arteria con el tipo de dominancia coronaria, los cuales pueden contribuir a un conocimiento más completo de una de las arterias del corazón con mayor relevancia clínica. El compromiso estructural y funcional de la arteria interventricular anterior, que se relaciona con enfermedad arterial coronaria responsable de una gran tasa de morbimortalidad, requiere de conocimientos detallados y precisos, a partir de la medicina basada en la evidencia científica ^4^. Por lo tanto, la información aquí descrita, que se ha fundamentado en una investigación descriptiva, aporta datos estructurales y clínicos que podrán ser tenidos en cuenta al momento de prestar servicios de salud a la persona con cardiopatía asociada con lesión arterial.

Esta información es crucial para el equipo de profesionales de la salud responsables de la valoración, el diagnóstico, el tratamiento y la rehabilitación de personas con enfermedad arterial coronaria. Este estudio también beneficia a los morfólogos y fisiólogos, porque aporta datos más detallados sobre los aspectos anatómicos y de la luz de la arteria interventricular anterior respecto no solo de su origen, sino de su trayecto, permeabilidad por cada uno de los tres segmentos valorados e intervenidos en hemodinamia, de lo que no hay reportes en otras investigaciones en población colombiana.

El objetivo de este artículo fue describir los aspectos anatómicos de la arteria interventricular anterior por técnica angiográfica; dichos aspectos incluyen el origen, el trayecto, la dominancia y la permeabilidad. Además, se describen algunas manifestaciones clínicas como isquemia, dolor precordial y enfermedad arterial coronaria, derivadas de las afecciones estructurales por alteración de su permeabilidad y de su relación con los tres tipos de dominancia comparativa por sexo.

## Materiales y métodos

Se desarrolló un estudio descriptivo de corte transversal, con 284 reportes de angiografías coronarias practicadas en el año 2022 en una clínica de gran complejidad del suroccidente colombiano. Se excluyeron los reportes de procedimientos realizados a personas nacidas fuera de Colombia, con antecedentes de cirugía de revascularización coronaria o con cateterismo coronario incompleto, quedando una muestra de 200 reportes angiográficos.

De cada reporte angiográfico, se obtuvieron variables cualitativas nominales como: sexo, antecedentes personales de salud que incluyeron dolor precordial y cardiopatía isquémica, dominancia coronaria y datos descriptivos de la arteria interventricular, como origen, trayecto y permeabilidad en sus segmentos S12, S13 y S14. Se consideró normal el origen anatómico común en el tronco de la arteria coronaria izquierda, de la circunfleja izquierda (*ramus circumflexus*) y la interventricular anterior (arteria descendente anterior izquierda). La permeabilidad coronaria se evaluó mediante la escala de flujo TIMI (*Thrombolysis in Myocardial Infarction*) y la dominancia coronaria se clasificó en derecha, izquierda o balanceada, siguiendo los criterios de Schlesinger.

Los datos se digitalizaron en una base sistematizada en Microsoft Excel para su tabulación y la obtención de porcentajes. Las imágenes angiográficas con variaciones anatómicas de la arteria interventricular anterior se analizaron con el *software* ACOM PC Lite de Siemens.

El desarrollo del presente estudio fue avalado por el comité institucional de revisión de ética humana de la Facultad de Salud de la Universidad del Valle, y la investigación fue clasificada sin riesgo conforme a lo definido en la Resolución 8430 de 1993 del Ministerio de Salud de Colombia. La información de los reportes angiográficos se manejó según lo dispuesto en la Resolución 1995 de 1999 del Ministerio de Salud de Colombia.

## Resultados

**Figura 1:**
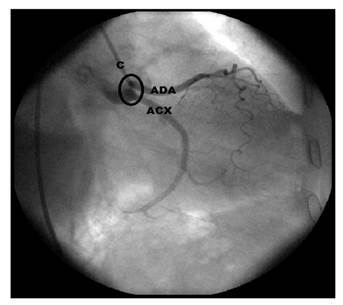
Imagen angiográfica con cateterismo. En el seno aórtico izquierdo no hay arteria coronaria izquierda y se identifican dos orificios independientes para dar origen a la arteria interventricular anterior y a la circunfleja izquierda.

Después de analizar los reportes de las 200 angiografías coronarias, se determinó que el 70,5 % correspondían a hombres y el 29,5 % a mujeres, con un promedio de edad de 64,22 (desviación estándar, DE: 11,22) años. El origen anatómico de la arteria interventricular anterior fue normal en el 99,05 % de la muestra, con una sola variación en una mujer; en ella, la arteria se originó en un orificio independiente localizado en el seno aórtico izquierdo y el tronco principal de la arteria coronaria izquierda estaba ausente, (figura 1).

El trayecto arterial más frecuente fue de tipo subepicárdico en el 98 % de los casos, con excepción de cuatro (2 %), tres hombres y una mujer, en quienes se identificó y reportó un puente muscular del segmento 13 o segmento intermedio de la arteria interventricular anterior. El 86 % de casos correspondió a dominancia coronaria derecha con origen normal de arteria interventricular anterior. Este tipo de dominancia fue la más frecuente en ambos sexos; sin embargo, la segunda dominancia más frecuente para hombres fue la izquierda, mientras que, para mujeres, fue del tipo balanceada. El caso de la arteria interventricular anterior con origen anómalo mostró dominancia izquierda.

La permeabilidad arterial fue normal en el 57 % del total de la muestra; se observaron alteraciones en la luz arterial en el 43 % de casos, con mayor frecuencia en los hombres. El segmento arterial más afectado fue el 13 o intermedio en hombres y el 12 o proximal en mujeres. El compromiso de un solo segmento arterial fue lo más frecuente, seguido del compromiso bisegmentario (cuadro 1).

**Cuadro 1:** Distribución de la permeabilidad de la arteria interventricular anterior según el sexo

Sexo	Permeabilidad		Segmento afectado*			Segmentos afectados**		
	Normal	Alterada	S12	S13	S14	1	2	3
Hombres	37,5 %	33 %	32	36	14	47	16	3
Mujeres	19,5 %	10 %	12	11	3	15	3	2

* Se refiere al número de casos con obstrucción en los segmentos 12, 13 y 14 de la arteria interventricular anterior.

** Se refiere al número de casos con obstrucción en 1, 2 o 3 segmentos de la arteria interventricular anterior.

Las causas más frecuentes de alteración de la permeabilidad correspondieron a placa arteriosclerótica, coágulo o trombo que, en algunos casos, fueron tratadas con análogos de los salicilatos, como el agrastat, con angioplastia y con implantación de endoprótesis vascular convencional o medicada. Los casos más graves se remitieron para presentarlos en una junta cardioquirúrgica. El 25 % de las personas, 40 hombres y 10 mujeres, presentaron dolor precordial. En el 40 %, se reportaron alteraciones ecocardiográficas, el 5 % presentó isquemia cardiaca y, el 59 %, alteración del trazado electrocardiográfico.

Cuatro casos presentaron alteración de tipo puente miocárdico en el trayecto de la arteria interventricular anterior: dos con dominancia derecha, uno con dominancia izquierda y otro con dominancia balanceada. Los dos casos con dominancia derecha presentaron dolor precordial; uno de los casos presentó oclusión de dos segmentos coronarios, con diagnóstico de infarto agudo del miocardio.

Al comparar la frecuencia del tipo de dominancia coronaria con la permeabilidad de la arteria interventricular, se encontró que, proporcionalmente, la codominancia o circulación balanceada es la que más frecuentemente se relaciona con lesiones obstructivas, más frecuentes en mujeres; la dominancia derecha es la que menos se relaciona con obstrucción (cuadro 2).

**Cuadro 2:** Relación entre el tipo de dominancia coronaria y permeabilidad de la arteria interventricular anterior

Dominancia coronaria	AIA permeable		AIA obstruida	
	Hombres	Mujeres	Hombres	Mujeres
Derecha	65	36	58	14
Izquierda	8	1	6	3
Balanceada	2	2	2	3

AIA: arteria interventricular anterior

En la mayoría de los casos, el dolor precordial fue concomitante con una lesión obstructiva de la arteria interventricular anterior, en hombres, del segmento 13 y, en mujeres, del 12. Según la irrigación coronaria, el dolor precordial se presentó principalmente en casos con dominancia derecha (cuadro 3).

**Cuadro 3:** Relación entre el dolor precordial con lesión segmentaria obstructiva de la arteria interventricular anterior y dominancia coronaria

Dolor precordial	Segmento AIA obstruido			Dominancia coronaria		
	S12	S13	S14	Derecha	Izquierda	Balanceada
Hombres	7	11	2	34	5	1
Mujeres	2	0	0	10	0	0

AIA: arteria interventricular anterior

## Discusión

Las variaciones anatómicas del origen de las arterias coronarias tienen una baja prevalencia en la población mundial. En Chile, Ugalde *et al*. desarrollaron un estudio prospectivo en 10.000 pacientes sometidos a angiografía coronaria y reportaron 1,29 % de anomalías en el origen de las arterias coronarias que, en el 0,8 % de los casos, involucraba a la arteria interventricular anterior con origen en el seno aórtico derecho, sin ningún otro tipo de variación para este vaso sanguíneo ^11^.

En España, Sarria *et al*. realizaron un estudio descriptivo con 1.180 angiotomografías y reportaron variaciones en el origen arterial coronario en el 2,2 %; el 15 % correspondió a ausencia del tronco coronario izquierdo, es decir, a un origen independiente de la arteria interventricular anterior y la circunfleja en el seno aórtico izquierdo, lo cual concuerda con la única variación de origen reportada en el presente estudio ^12^.

Kosar *et al*. evaluaron 700 reportes de angiotomografía coronaria con un porcentaje de variación del origen del 3,9 %; en el 0,4 % de estos casos, la arteria interventricular anterior se originó en un orificio independiente en el seno aórtico izquierdo ^13^.

Estos estudios referenciados mostraron hallazgos similares a los de la presente investigación, la cual mostró una muy pequeña prevalencia de variación del origen anatómico de las coronarias, que involucró la arteria interventricular anterior con origen independiente en el seno aórtico izquierdo. El trayecto normal de la interventricular anterior, al igual que las demás ramas de las arterias coronarias, es de tipo subepicárdico, es decir, entre el epicardio y el miocardio, donde se encuentran rodeadas por tejido conjuntivo adiposo, denominado grasa subepicárdica. Las variaciones del trayecto arterial coronario más frecuentes, son de tipo intramiocárdico o puente miocárdico, es decir, la arteria penetra en el espesor del músculo estriado cardiaco y lo atraviesa en alguno de sus segmentos o, incluso, en todo su recorrido ^14^. Los reportes angiográficos del presente estudio no refirieron el grado de profundidad del puente miocárdico; sin embargo, la única arteria afectada fue la interventricular anterior, tal como lo sugiere Pérez ^15^.

En Colombia, Ballesteros *et al*. realizaron un estudio descriptivo observacional en 154 corazones, que fueron disecados e inyectados con resina en sus arterias coronarias. Encontraron 92 puentes miocárdicos en diferentes segmentos arteriales de 62 corazones. La arteria con mayor cantidad de puentes fue la interventricular anterior, en sus segmentos 12 y 13 ^16^. En Brasil, Sousa *et al*. disecaron la arteria interventricular anterior de 30 corazones humanos y reportaron puentes miocárdicos en el 46,7 %, los cuales afectaban principalmente a los segmentos 13 y 12 ^17^.

Al comparar los hallazgos de puentes musculares reportados en estos dos informes con los del presente estudio, hay una diferencia importante. Es probable que la identificación de los puentes miocárdicos mediante procedimientos angiográficos se dificulte más que con la disección directa, sobre todo cuando se trata de puentes parcialmente tunelizados o delgados. En un estudio descriptivo por medio de angiotomografía computarizada de 393 pacientes, De Agustín *et al*. encontraron 86 puentes en 82 pacientes, en el 87,2 % de los cuales se involucró a la arteria interventricular anterior; los puentes miocárdicos se asociaron con miocardiopatía ^18^.

En los estudios anatómicos se describe la dominancia coronaria derecha como la más frecuente (85 %), lo que concuerda con el 86 % de los reportes del presente estudio. También, concuerda con un estudio descriptivo directo en población mestiza colombiana, en el cual se encontró dominancia derecha en el 83,7 % de los casos, seguida de circulación balanceada en el 9,2 % ^19^. Según Ballesteros *et al*., el 56 % de los puentes miocárdicos se presentó en casos de circulación balanceada y, el 39,3 %, en casos de dominancia derecha; la arteria interventricular anterior fue la más comprometida ^16^.

Estos hallazgos coinciden con los del presente estudio en que la arteria interventricular anterior es la que muestra mayor cantidad de puentes miocárdicos; sin embargo, la dominancia que más se asoció con el hallazgo de puentes fue la derecha. La influencia de la dominancia coronaria izquierda en el dolor precordial asociado con isquemia en la cara anterior del ventrículo izquierdo y el ápex cardiaco, irrigados por la arteria interventricular anterior, tiene valor clínico para el pronóstico a largo plazo del paciente con este tipo de cardiopatía ^20^.

El flujo sanguíneo en la arteria interventricular anterior es vital para el suministro de oxígeno y nutrientes al fascículo auriculoventricular o haz de His, y al miocardio apical y el de la cara anterior del ventrículo izquierdo; por tanto, la alteración de su permeabilidad se asocia con cardiopatía isquémica, infarto del miocardio y alteración de la conducción eléctrica en los ventrículos. La escala TIMI se utiliza en cardiología intervencionista al evaluar la permeabilidad de las arterias coronarias y determinar el flujo sanguíneo que llega al músculo cardíaco. Su puntaje va de 0 a 3: 0 es ausencia de flujo; 1, flujo mínimo o inapreciable; 2, flujo lento pero sostenido; y 3, flujo normal ^21^. En la revisión de Dattoli *et al*., la angiografía demostró alteración de la permeabilidad coronaria con compromiso de la arteria interventricular anterior en el 71,5 % de los casos, y dicha lesión se asoció con infarto agudo de miocardio en el 6 al 12 % de los pacientes y que dichos hallazgos suelen ser cada vez más frecuentes en personas menores de 45 años con antecedentes de tabaquismo y dislipidemia ^22^.

Algunas de las indicaciones para la angiografía coronaria son los cambios electrocardiográficos del ST, el dolor precordial y la alteración de la prueba de esfuerzo. No obstante, no todos los procedimientos angiográficos en este tipo de pacientes indican alteración de la permeabilidad coronaria. Las personas con dolor precordial e isquemia miocárdica, sin obstrucción de las arterias coronarias, se pueden asignar a otros grupos, uno con infarto miocárdico, denominado MINOCA (*Myocardial Infarction with Non-Obstructive Coronary Arteries*), y otro con angina e isquemia, llamado INOCA (*Ischemia with Non-Obstructive Coronary Arteries*) ^23^. Estas asociaciones son importantes; en el presente estudio, el 16 % de las personas con alteraciones electrocardiográficas no presentaron ninguna lesión obstructiva en la arteria interventricular anterior.

Pérez-Riera *et al*. describieron el caso de una mujer de 73 años con antecedentes de dislipidemia, prediabetes e hipertensión arterial, con signos de taquicardia sinusal y extrasístole ventricular en el electrocardiograma; su angiotomografía coronaria demostró una lesión obstructiva del S12 de la arteria interventricular anterior. Se implantó una endoprótesis vascular (*stent*) medicada y la paciente mejoró. Esto es un claro indicador de la importancia de la arteria interventricular anterior en el sistema de conducción cardiaco por la irrigación proporcionada al fascículo aurículo-ventricular ^8^.

Montero-Cabezas *et al*. presentaron dos casos de enfermedad obstructiva de la arteria interventricular anterior, precedida por depresión del segmento ST en el electrocardiograma, aunque la indicación de procedimientos de intervencionismo e, incluso, de cirugía de revascularización de urgencia, se asocia con elevación de dicho segmento ST en personas con dolor precordial y oclusión coronaria. Por lo tanto, no siempre el trazado del electrocardiograma se asocia con con alteración de la permeabilidad coronaria ^4^.

Las variaciones anatómicas del origen de la arteria interventricular anterior fueron poco frecuentes; en el presente estudio, se presentó en el 0,5 % de los casos. Según los conocimientos actuales, ocupan el cuarto lugar entre el grupo de variaciones coronarias. En primer lugar, está la arteria coronaria con origen en el seno contralateral; en segundo lugar, la arteria coronaria única; y en tercer lugar, la arteria coronaria derecha originada en la interventricular anterior.

El trayecto normal de las arterias coronarias es subepicárdico; es decir, entre el epicardio y miocardio. Sin embargo, los puentes musculares se consideran la principal variación del trayecto coronario que, en el caso de la arteria interventricular anterior, representó el 2 %; en otros estudios, este tipo de variante del trayecto arterial se reporta hasta en el 47 % de los casos.

La dominancia coronaria derecha presentó la mayor asociación con el dolor precordial. La dominancia en la irrigación influyó sobre la presencia de puentes musculares, pero sí se correlacionó con alteración de la permeabilidad en mujeres con circulación balanceada; la dominancia derecha fue la que con menor frecuencia se relacionó con obstrucción de la arteria interventricular anterior.

Los hallazgos del presente estudio no difirieron de los de otros reportes de otras partes del mundo, respecto a prevalencias de origen anatómico normal y variante, dominancia coronaria y alteración de la permeabilidad. Se considera novedosa en este estudio, la descripción de cada uno de los tres segmentos de la arteria interventricular anterior, que, finalmente, son los que se valoran clínicamente mediante hemodinamia y cardiología intervencionista, para la toma de decisiones en pacientes con cardiopatía isquémica.

## References

[1] Corcho D, Urrego J, Suárez C, Barbosa J, Vargas C, Suárez E Indicadores básicos de salud 2022. Situación en Colombia.

[2] Moscucci M. (2013). Grossman & Baim’s Cardiac catheterization, angiography, and intervention.

[3] Bastarrika G, Alonso A, Azcárate P, Castaño S, Pueyo J, Alegría E. (2008). Anatomía normal, variantes anatómicas y anomalías del origen y trayecto de las arterias coronarias por tomografía computarizada multicorte. Radiología.

[4] Montero-Cabezas J, van der Kley F, Karalis I, Schalij M. (2015). Oclusión aguda de la arteria descendente anterior proximal con patrón electrocardiográfico inusual: no todo es ascenso del ST. Rev Esp Cardiol.

[5] Rodríguez Y, Alves A, Pessuti F, Weinert M. (2015). Fístula entre la arteria coronaria descendente anterior y el tronco de la arteria pulmonar: Reporte de un caso clínico. Gac Médica Boliv.

[6] Kasprzak J, Al-Shaibi K, Ahmed W, Chamsi-Pasha H, Nosir Y. (2008). Origen anómalo de la arteria coronaria izquierda: inusual coincidencia de arteria pulmonar como origen de la arteria descendente anterior y arteria circunfleja originada en el seno derecho de Valsalva. Rev Esp Cardiol.

[7] Martínez-Cano C, Romero-Seguro S, Ramírez-Ramos C, Echeverry D, Bolívar-Mejía L. (2022). Anomalous origin of the anterior descending artery. Rev Colomb Cardiol.

[8] Pérez Riera G, Barbosa Barros R, Daminello Raimundo R, de Abreu L, Nikus K. (2019). Transient left septal fascicular block and left anterior fascicular block as a consequence of proximal subocclusion of the left anterior descending coronary artery. Ann Noninvasive Electrocardiol.

[9] Latarjet M, Ruiz Liard A. (2019). Anatomía humana.

[10] Drake R, Vogl W, Mitchell A. (2020). Gray, Anatomía para estudiantes.

[11] Ugalde H, Ramírez A, Ugalde D, Farias E, Silva A. (2010). Nacimiento anómalo de las arterias coronarias en 10.000 pacientes adultos sometidos a coronariografía. Rev Med Chile.

[12] Sarria S, Arteche E, Certo M, Fernández G. (2007). Valoración mediante TC multidetector de las variantes anatómicas en las arterias coronarias. Colomb Med.

[13] Koçar P, Ergun E, Õztürk C, Koçar U. (2009). Anatomic variations, and anomalies of the coronary arteries: 64-slice CT angiographic appearance. Diagnostic Interv Radiol.

[14] Sternheim D, Power D, Samtani R, Kini A, Fuster V, Sharma S. (2021). Myocardial bridging: Diagnosis, functional assessment, and management: JACC State-of-the-art review. J Am Coll Cardiol.

[15] Pérez G. Puente muscular de la arteria descendente anterior.

[16] Ballesteros-Acuña LE, Ramírez-Aristeguieta LM, Saldarriaga B. (2008). Descripción morfológica e implicaciones clínicas de puentes miocárdicos: un estudio anatómico en colombianos. Arq Bras Cardiol.

[17] Sousa C, Soares F, Buarque L, Da Rocha A, Alencar R, Olave E. (2006). Aspectos anatómicos y biométricos de los puentes de miocardio y sus relaciones con la arteria interventricular anterior y venas adyacentes. Int J Morphol.

[18] De Agustín J, Marcos P, Fernández C, Bordes S, Feltes G, Almería C (2012). Puente miocárdico evaluado mediante tomografía computarizada multidetectores: posible causa del dolor torácico en pacientes más jóvenes con baja prevalencia de dislipemia. Rev Esp Cardiol.

[19] Ballesteros L, Corzo E, Saldarriaga B. (2007). Determinación de la dominancia coronaria en población mestiza colombiana. Un estudio anatómico directo. Int J Morphol.

[20] Abu E, Castiñeira M, González V, Raposeiras S, Abumuaileq R, Peña C (2016). Dominancia coronaria y pronóstico a largo plazo de los pacientes con infarto de miocardio con elevación del segmento ST tratado con angioplastia primaria. Rev Esp Cardiol.

[21] Pérez A, Fernández F, Cuellas J, Michael C. (2006). Coronariografía: más allá de la anatomía coronaria. Rev Esp Cardiol.

[22] Dattoli C, Jackson C, Gallardo A, Gopar R, Araiza D, Arias A. (2021). Acute myocardial infarction: Review on risk factors, etiologies, angiographic characteristics, and outcomes in young patients. Arch Cardiol Mex.

[23] Cohen H, Iglesias R, Duronto E, Lescano A, Campisi R, Deviggiano A (2020). isquemia miocárdica sin lesiones coronarias obstructivas: Minoca-Inoca. Revisión para la toma de decisiones. Medicina.

